# Comparative analysis of gut fungal composition and structure of the yaks under different feeding models

**DOI:** 10.3389/fvets.2023.1193558

**Published:** 2023-06-15

**Authors:** Yanbin Zhu, Yangji Cidan, Guangming Sun, Xin Li, Muhammad Akbar Shahid, Zhaxi Luosang, Zhaxi Suolang, Lang Suo, Wangdui Basang

**Affiliations:** ^1^Institute of Animal Husbandry and Veterinary Medicine, Tibet Academy of Agriculture and Animal Husbandry Sciences, Lhasa, China; ^2^Linzhou Animal Husbandry and Veterinary Station, Lhasa, China; ^3^Lanzhou Veterinary Research Institute, Chinese Academy of Agricultural Sciences, Lanzhou, China; ^4^Key Laboratory of Animal Genetics and Breeding on Tibetan Plateau, Ministry of Agriculture and Rural Affairs, Lhasa, China; ^5^Department of Pathobiology, Faculty of Veterinary Sciences, Bahauddin Zakariya University, Multan, Pakistan

**Keywords:** yak, wild, Qinghai-Tibet plateau, gut microbiota, gut fungal community

## Abstract

The yaks that inhabit the Tibetan plateau are a rare breed that is closely related to local economic development and human civilization. This ancient breed may have evolved a unique gut microbiota due to the hypoxic high-altitude environment. The gut microbiota is susceptible to external factors, but research regarding the effects of different feeding models on the gut fungal community in yaks remains scarce. In this study, we compared and analyzed the composition and variability of the gut fungal community among wild yaks (WYG), house-feeding domestic yaks (HFG), and grazing domestic yaks (GYG). The results revealed that Basidiomycota and Ascomycota were the most preponderant phyla in the gut fungal community, regardless of feeding models. Although the types of dominant fungal phyla did not change, their abundances did. Intergroup analysis of fungal diversity showed that the Shannon and Simpson indices of WYG and GYG were significantly higher than those of HFG. Fungal taxonomic analysis showed that there were 20 genera (*Sclerostagonospora* and *Didymella*) that were significantly different between WYG and GYG, and 16 genera (*Thelebolus* and *Cystobasidium*) that were significantly different between the WYG and HFG. Furthermore, the proportions of 14 genera (*Claussenomyces* and *Papiliotrema*) significantly decreased, whereas the proportions of eight genera (*Stropharia* and *Lichtheimia*) significantly increased in HFG as compared to GYG. Taken together, this study indicated that the gut fungal composition and structure differ significantly between yaks raised in different breeding groups.

## Introduction

It is widely known that the gut microbiota is a complex micro-ecosystem involving a large number of different types of microorganisms, including bacteria, fungi, and viruses (1–3). Studies have shown that the gut microbiota can provide the host with nutrients and beneficial metabolites, such as amino acids, vitamins, and short-chain fatty acids, by fermenting sugars and carbohydrates (4–6). These metabolites play important roles in host health, immunity, intestinal homeostasis, and intestinal barrier (7, 8). Similar to the gut bacterial community, the gut fungal community is also an important component of the gut microbiota, which plays vital roles in host health by improving gut functions (9–12). Early investigations indicated that the gut fungal community could induce significant shifts in the gut bacterial structure and shape the gut microbiota during early life (13). The gut fungal community may also play a role in the maturation of the host immune system by interacting with the gut bacterial community to produce strong local and systemic immune responses (13, 14). Furthermore, some fungi are considered to be intestinal probiotics due to their potential role in alleviating inflammation, inhibiting pathogenic bacteria, degrading cellulose, and regulating digestion (15–17). For instance, administration of *Candida kefyr* has been demonstrated to alleviate gastrointestinal inflammation by changing the gut microbiota (18). Additionally, some fungi can synthesize and release neurotransmitters such as norepinephrine and histamine (19, 20). However, gut microbial homeostasis is easily affected by external factors such as age, sex, diet, and disease (21, 22). Moreover, recent studies have indicated that feeding methods, altitude, and habitat environment are also important driving forces for the development of gut microbiota (23, 24).

Yak is a rare species of cattle of the Tibetan plateau (above 3,000 m), which is characterized by adapting to high, cold, and oxygen-deficient environments (25). Statistically, approximately 90% of the world's yaks live in the Sichuan, Qinghai, Tibet, and Gansu provinces of China (26, 27). Yaks are also an important source of milk and meat products for local herdsmen and play an important role in economic development. Given the importance of yaks on the Tibetan plateau, any factors that threaten the health and development of this breed may lead to enormous economic losses. Previous studies indicated that cold and hypoxic environments could cause changes in the gut microbial structure (28–31). Therefore, the altitude hypoxia environments of the Tibetan Plateau may induce the accumulation of special gut microbiota in yaks compared with animals living in plains. Indeed, several studies have reported the unique composition and diversity of the gut microbiota in yaks (32, 33).

In the past, yaks were mainly raised in the open and were easily affected by the external environment. For instance, changeable weather and nutritional deficiencies can cause low production efficiency and a high disease rate of yaks (34, 35). To improve the productivity of yaks, a combination of free grazing and barn feeding is also implemented in some areas. Furthermore, there are still some wild yaks in the same area of the Tibetan Plateau. Although these yaks are the same species, they may have evolved specific microbial communities to adapt to different farming methods. A previous study has demonstrated significant differences in the gut bacterial community of yaks under different feeding models (36, 37). However, until now, little research has been conducted on the gut fungal community of yaks. Therefore, the aim of our study was to evaluate the composition and variability of the gut fungal community of yaks under different feeding models.

## Materials and methods

### Sample acquisition

In this study, fecal samples of grazing domestic yaks (GYG) and wild yaks (WYG) were obtained from Shuanghu County and Chang Tang Nature Reserve, China. The average altitude of this area exceeds 5,000 meters and is characterized by high temperatures, low precipitation, and strong wind speed in July and August. Moreover, the zonal vegetation includes *Austrostipa pubescens* as the dominant vegetation. We also collected fecal samples from house-fed domestic yaks (HFG), which were mainly fed green hay, to explore the changes in the gut fungal community under different feeding models. All samples were collected in July 2022, and each group contained five animals. Fecal samples from yaks of different feeding models were collected using dung samplers. Freshly rectal feces were selected and sub-sampled (approximately 100 g) from the central portion to minimize contamination from bedding and flooring. Subsequently, fecal samples from yaks of different feeding models were labeled (GYG: GYG1, GYG2, GYG3, GYG4, and GYG5; WYG: WYG1, WYG2, WYG3, WYG4, and WYG5; HFG: HFG1, HFG2, HFG3, HFG4, and HFG5) and placed at −80°C for further analysis.

### DNA extraction and high-throughput sequencing

Fungal DNA was extracted from homogenized intestinal contents using a QIAamp DNA Mini Kit (QIAGEN, Hilden, Germany), based on the manufacturer's protocol. Subsequently, we amplified the ITS2 regions using primers (ITS5F: GGAAG TAAAAGTCGTAACAAGG and ITS2R: GCTGCGTTCTTCATCGA TGC) and synthesized them as per conserved regions. PCR amplification procedures were determined according to previous research (38–40). After purification and fluorescence quantification of the amplified products, the sequencing library was constructed using the TruSeq Nano DNA LT Library Prep Kit (Illumina, CA, USA). The initial libraries were subjected to sequence end repair, enrichment, and purification to improve their quality. The final libraries that passed the quality assessment were used to perform paired-end sequencing using a MiSeq sequencing machine. Raw data from the amplicon sequencing were further processed and modified using QIIME software (Qiime1.9.1) due to the presence of questionable sequences such as unqualified, short, mismatched, and chimera sequences. After quality inspection and filtering, the effective reads were applied for OTU clustering based on 97% similarity. Fungal OTU representative sequences were taxonomically classified using RDP Classifier v.2.2 based on the UNITE database (39, 41). Meanwhile, the Venn diagram was also generated to observe the common and unique OTUs among the groups. To study the variation in gut microbial diversity and abundance, we calculated multiple alpha diversity indices based on the number of OTUs in each sample using QIIME software (Qiime1.9.1). Moreover, PCoA plots reflecting beta diversity were also generated using QIIME software (Qiime1.9.1) to further compare and analyze the differences in gut microbial principal components. Statistical analysis of the data was conducted using R (v3.0.3) and GraphPad Prism (version 8.0c). LEfSe and meta-statistics analyses were employed to detect differential fungal taxa. The *p*-values (means ± SD) of < 0.05 were considered statistically significant.

## Results

### Sequencing data analysis

**Figure 1:**
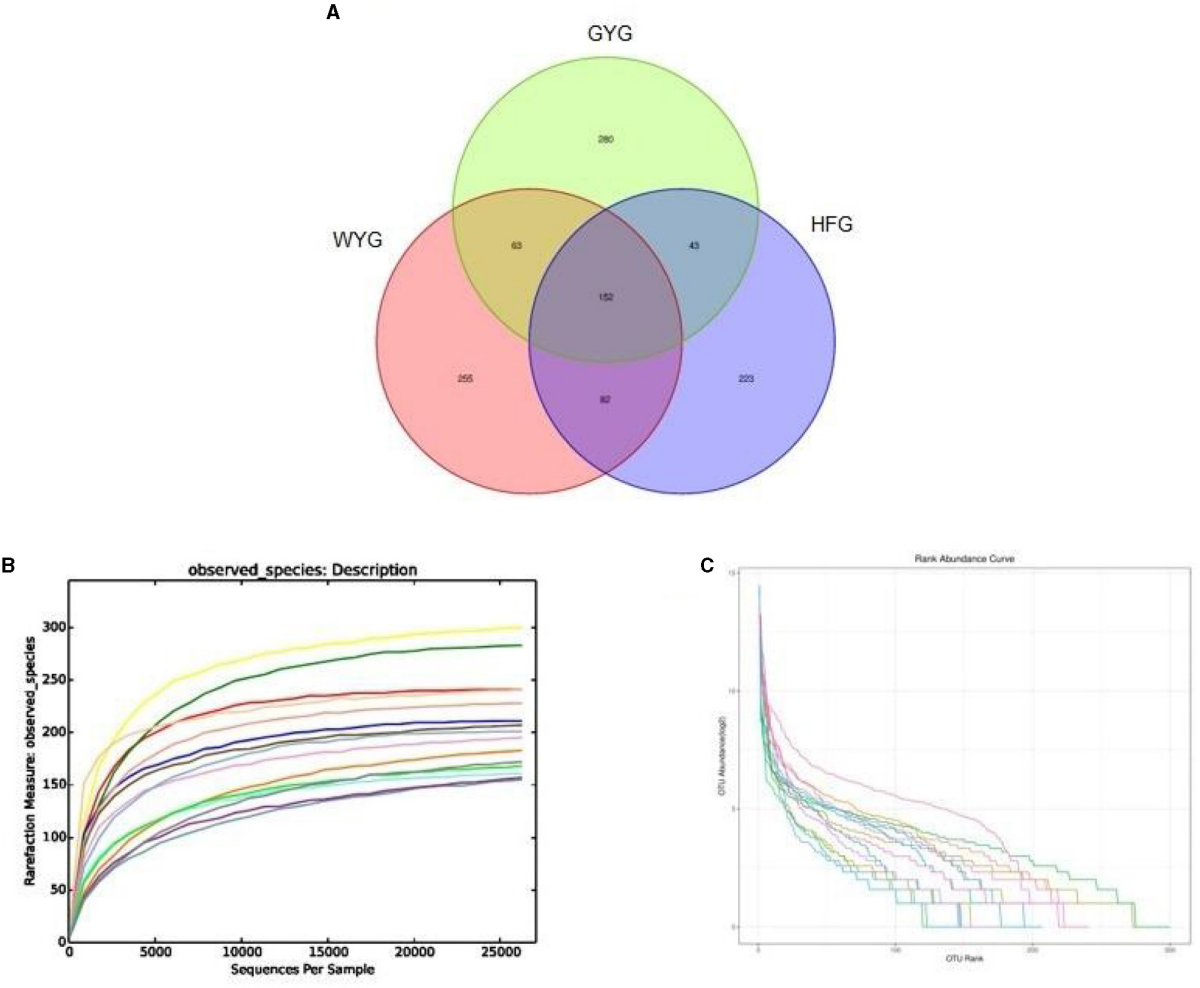
Sequencing data analysis and OTU distribution. Each colored curve in the rarefaction and rank abundance curves represents one sample. **(A)** The number of OTUs is indicated by different colored areas, and the middle area indicates the number of shared OTUs. **(B)** Rarefaction curve. **(C)** Rank abundance curve.

In this research, high-throughput sequencing generated 576,964 (WYG = 182,234, HFG = 193,448, and GYG = 201,282) valid sequences from the three groups with an average of 38,464 (ranging from 30,248 to 45,539) reads per sample (Appendix A). The high-quality sequences were clustered, and a total of 1,098 OTUs were identified, ranging from 500 to 552 OTUs per group (Figure 1A). Additionally, the numbers of unique OTUs in the WYG, HFG, and GYG were 255, 223, and 280, respectively. Notably, we also found 152 core OTUs in the three groups, which were not affected by the feeding models. To assess sequencing depth and evenness, we also generated rarefaction and species rank curves. The results indicated that the curves of all samples exhibited a saturation trend, suggesting the adequacy and reliability of the sequencing (Figures 1B, C).

### Comparative analysis of the gut fungal community in yaks with different feeding models

**Figure 2:**
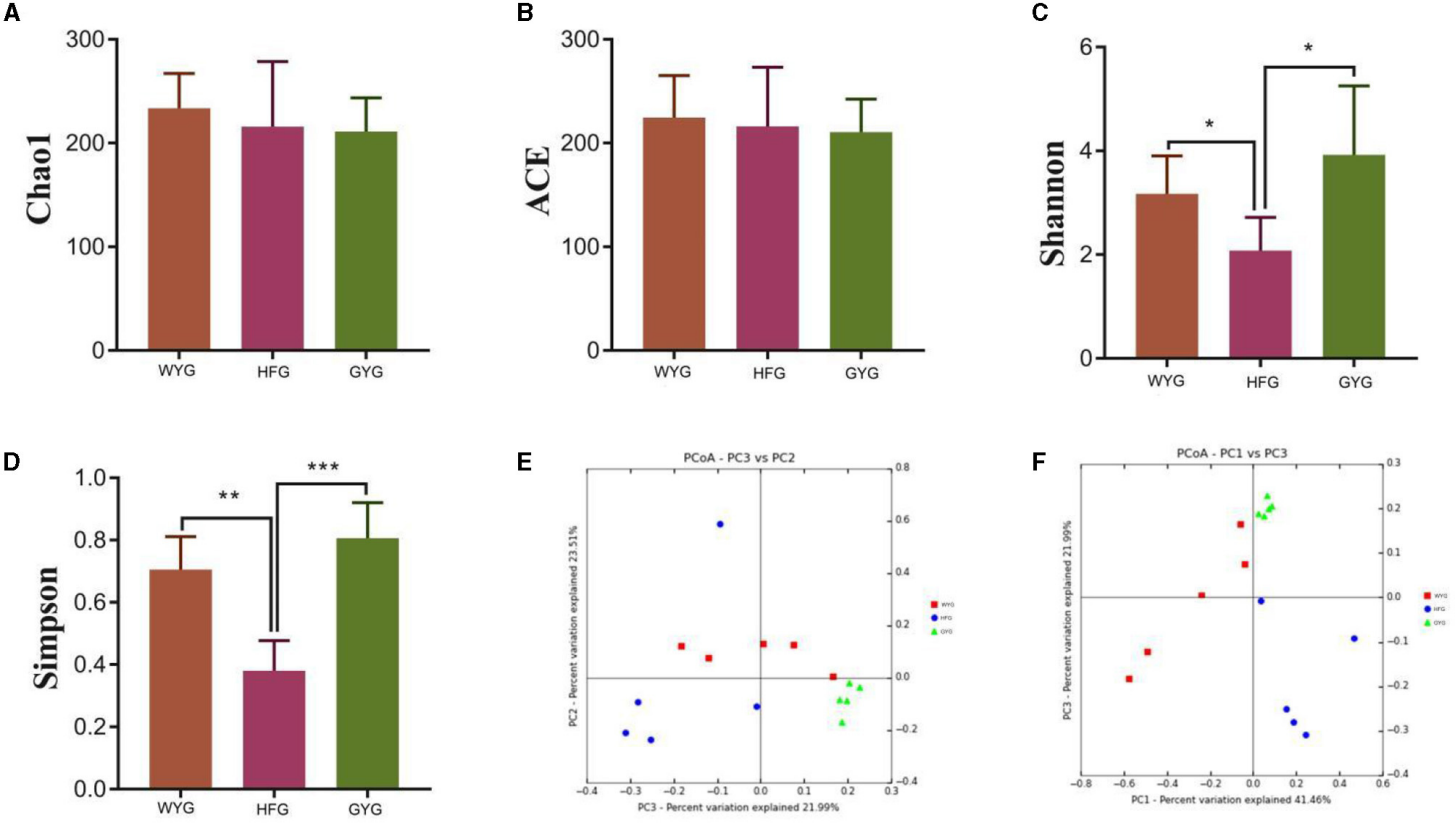
Comparative analysis of gut fungal diversity in yaks under different feeding models. The four indices including Chao1, ACE, Shannon, and Simpson were used to assess alpha diversity. **(A)** Chao1 index. **(B)** ACE index. **(C)** Shannon index. **(D)** Simpson index. **(E, F)** Gut fungal beta diversity was assessed by PCoA plots. Data are presented as means ± SD. *P < 0.05, **P < 0.01, and ***P < 0.001.

To explore the effects of different feeding models on the gut fungal alpha diversity of yaks, we calculated four indices, including Chao1, ACE, Simpson, and Shannon indices (Figures 2A–D). The Chao1 and ACE indices of the gut fungal community, from high to low, are WYG, HFG, and GYG. Moreover, the GYG had the highest Simpson and Shannon indices, followed by WYG and HFG. There were statistically distinct differences in Simpson (0.71 ± 0.11 vs. 0.38 ± 0.097, *P* < 0.01) and Shannon (3.17 ± 0.73 vs. 2.08 ± 0.64, *P* < 0.05) indices, whereas the Chao1 (233.69 ± 33.47 vs. 215.94 ± 62.69, *P* > 0.05) and ACE (224.71 ± 40.33 vs. 216.11 ± 57.06, *P* > 0.05) indices were not significantly different between the WYG and HFG. Similarly, we also found that the Simpson (0.80 ± 0.11 vs. 0.38 ± 0.097, *P* < 0.001) and Shannon (3.92 ± 1.33 vs. 2.08 ± 0.64, *P* < 0.05) indices of the GYG were significantly higher than those of the HFG, while there was no difference in the Chao1 (211.04 ± 32.59 vs. 215.94 ± 62.69, *P* > 0.05) and ACE (210.71 ± 31.78 vs. 216.11 ± 57.06, *P* > 0.05) indices. The comparative analysis between WYG and GYG indicated that there were no significant differences in the four indices. These results indicated that the gut fungal diversity in WYG and GYG was significantly higher than that in HFG, while there was no difference in the gut fungal abundance among WYG, HFG, and GYG. The results of the beta analysis showed that the samples of different groups were separated from each other, indicating significant differences in the principal components of the gut fungal community (Figures 2E, F).

### Composition and differences of the gut fungal community in yaks with different feeding models

**Figure 3:**
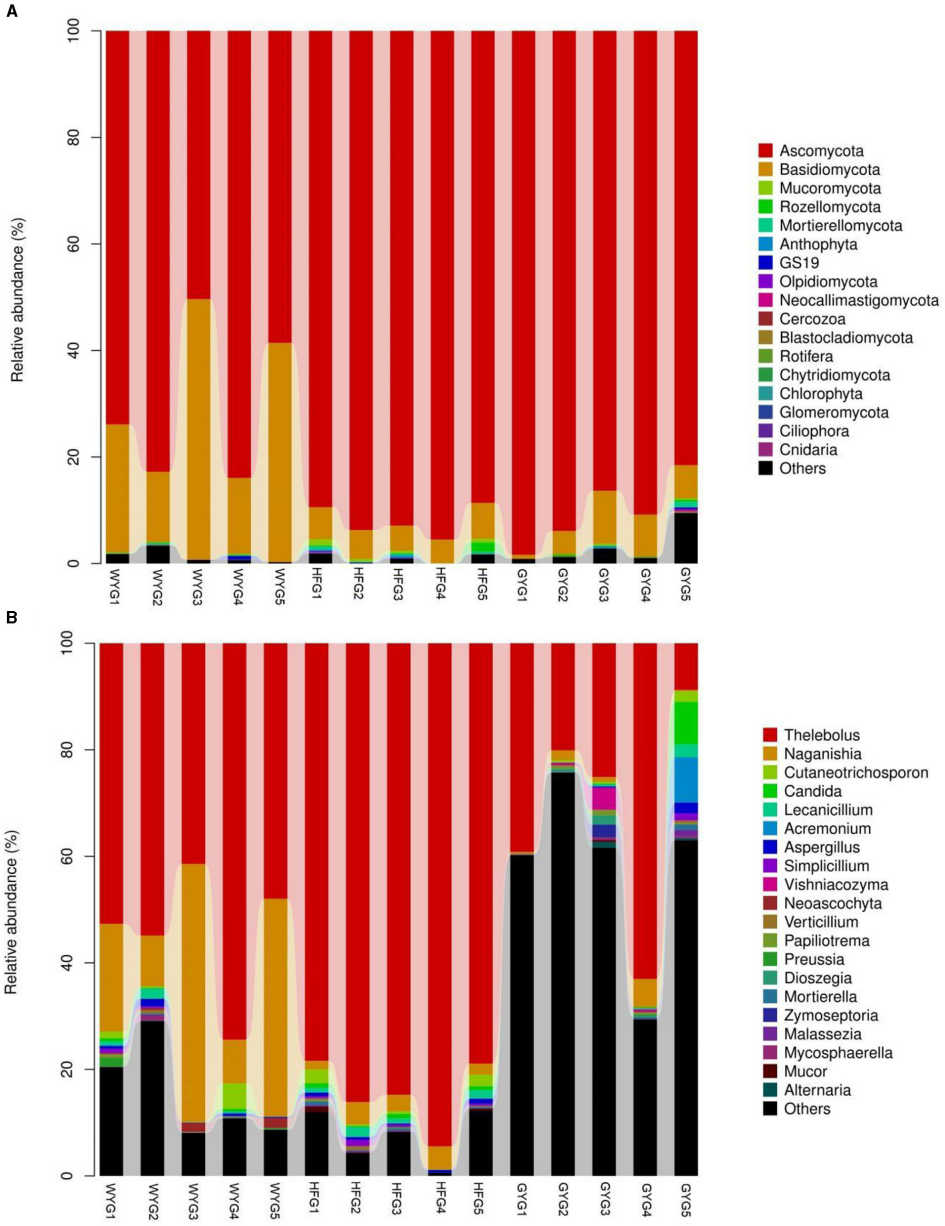
The abundance of dominant fungal phyla and genera in the gut fungal community of yaks under different feeding models. **(A)** The abundance of dominant fungal phyla. **(B)** The abundance of dominant fungal genera. The abundance of different fungal phyla or genera is represented by different colors and the height of the histogram.

**Figure 4:**
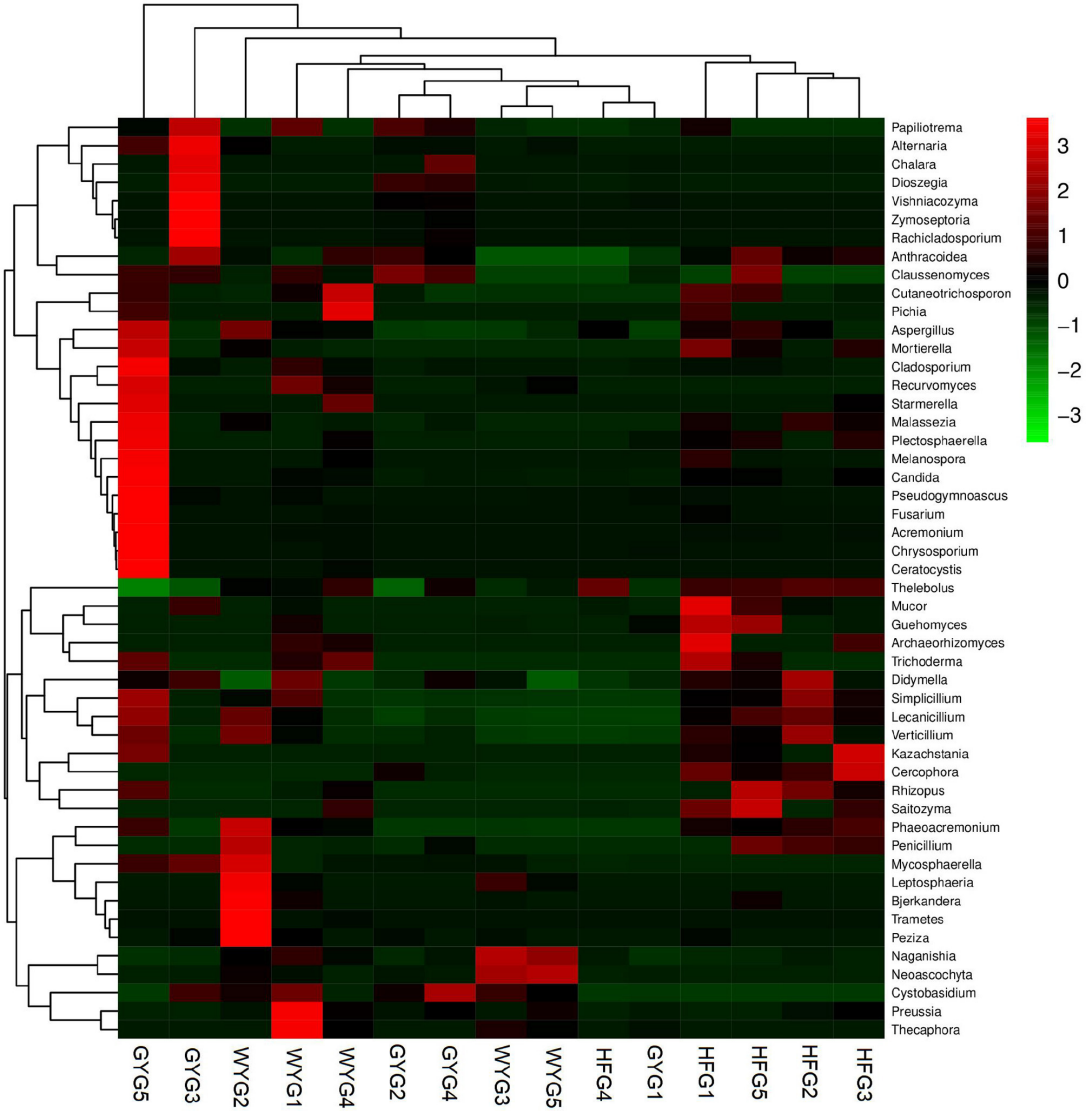
Heatmap of the abundant fungal genera in the WYG, HFG, and GYG. The abundance of different fungal genera is represented by different colors.

The phyla Ascomycota (69.90, 92.02, and 90.18%) and Basidiomycota (28.33, 5.47, and 5.86%) were abundant in the WYG, HFG, and GYG, accounting for more than 95% of the total fungal composition, respectively (Figure 3A). Moreover, other phyla, such as Mucoromycota (0.058, 0.57, and 0.11%), Rozellomycota (0.078%, 0.39%, and 0.19%), Mortierellomycota (0.058, 0.26, and 0.21%), Anthophyta (0.011, 0.13, and 0.082%), GS19 (0.14, 0.012, and 0.067%), Olpidiomycota (0.00, 0.086, and 0.02%), Neocallimastigomycota (0.017, 0.0068, and 0.069%), Cercozoa (0.012, 0.00, and 0.059%), Blastocladiomycota (0.00, 0.00, and 0.044%), and Rotifera (0.022, 0.0053, and 0.012%), were detected in WYG, HFG, and GYG in low abundances. *Thelebolus* (54.27%), *Naganishia* (25.43%), and *Cutaneotrichosporon* (1.29%) were the most predominant genera in the WYG (Figure 3B). Moreover, the dominant genera found in the HFG were *Thelebolus* (84.52%), *Naganishia* (3.08%), *Cutaneotrichosporon* (1.11%), and *Lecanicillium* (1.03%). The fungal genera with an abundance of more than 1% in the GYG were *Thelebolus* (31.22%), *Naganishia* (1.71%), *Candida* (1.65%), *Acremonium* (1.72%), and *Vishniacozyma* (1.00%). Furthermore, the gut fungal compositions and changes in WYG, HFG, and GYG could also be observed through a visualized clustering heatmap (Figure 4).

**Figure 5:**
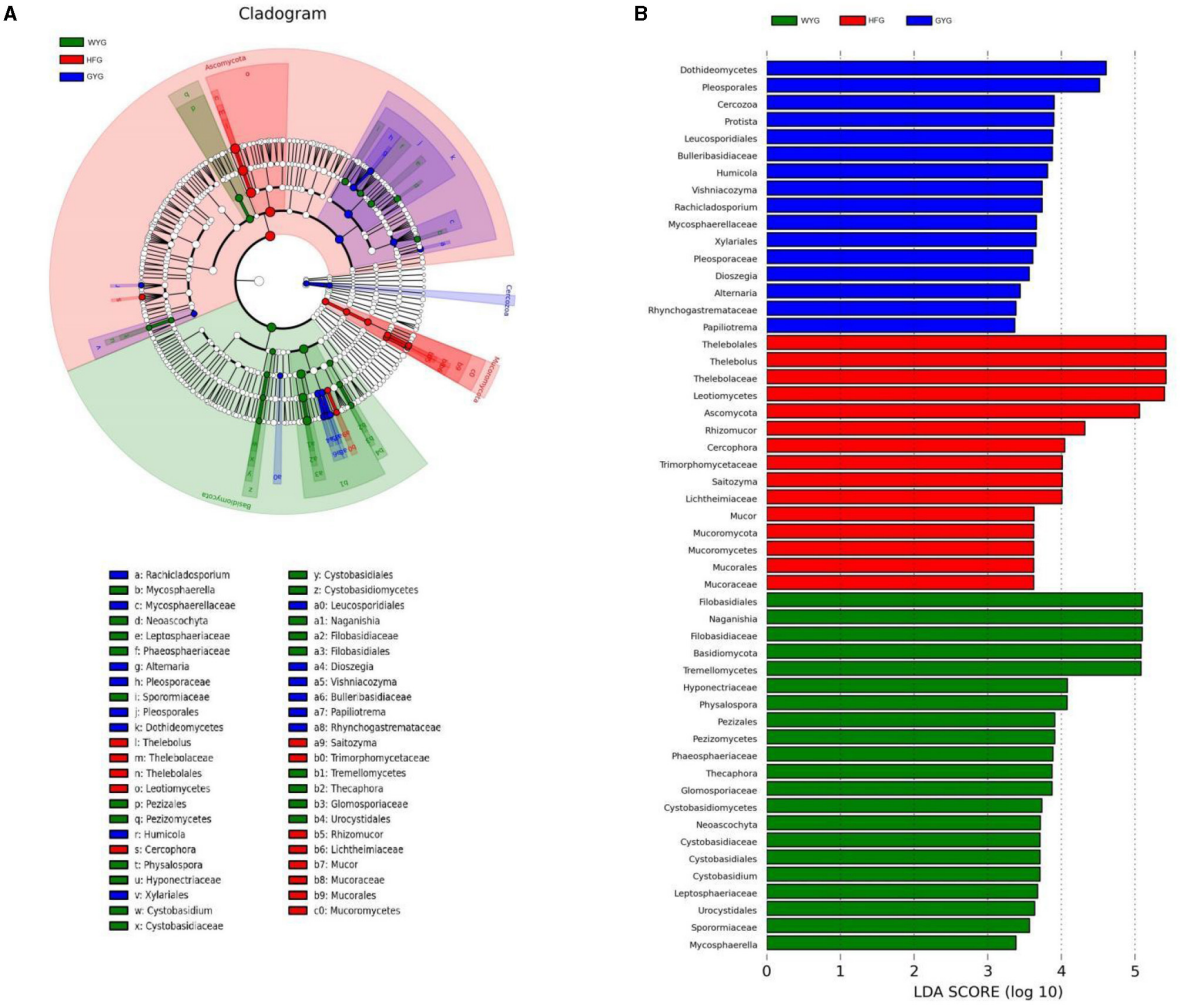
Detection of differentially abundant taxon using LEfSe and LDA scores. **(A)** Differential fungi and phylogenies are represented by a cladogram. **(B)** Differential fungi of yaks under different feeding models are represented by LDA scores.

We also conducted meta-statistics analysis to identify differences in the gut fungal community at different taxonomic levels. At the phyla level, the HFG indicated significantly higher levels of Ascomycota and Mucoromycota, while the WYG enriched for Basidiomycota (Table 1). A comparison of the GYG and WYG showed an obvious increase in the levels of Ascomycota and Cercozoa and a distinct reduction in the level of Basidiomycota. Additionally, the abundance of Cercozoa in GYG was significantly more dominant than that in HFG, whereas that of Mucoromycota was lower. Compared with the HFG, the fungal community in the WYG displayed an obvious increase in the relative abundances of *Cystobasidium, Naganishia, Sclerostagonospora, Physalospora, Neoascochyta, Recurvomyces, Schizonella, Orpinomyces, Herpotrichia, Sphaerulina*, and *Podospora*, while *Thelebolus, Cercophora, Stropharia, Lichtheimia*, and *Rhizopus* decreased dramatically. The GYG indicated dramatically higher proportions of *Didymella, Claussenomyces, Anthracoidea, Neoascochyta, Papiliotrema, Mastigosporium, Dioszegia, Heyderia, Ramularia, Cladonia, Urocystis*, and *Parapenidiella*, whereas the WYG was dramatically enriched for *Naganishia, Sclerostagonospora, Kurtzmanomyces, Thermoascus, Physalospora, Herpotrichia, Panaeolus*, and *Archaeorhizomyces*. Moreover, the abundances of *Cystobasidium, Claussenomyces, Papiliotrema, Mycosphaerella, Neoascochyta, Mastigosporium, Dioszegia, Urocystis, Humicola, Physalospora, Heyderia, Sphaerulina, Ramularia*, and *Cladonia* in the GYG were significantly preponderant compared to the HFG, while the abundances of *Stropharia, Lichtheimia, Saitozyma, Penicillium, Pyrenochaetopsis, Thermomyces, Knufia*, and *Melanocarpus* were lower. Given that this discriminant analysis did not distinguish the predominant taxon, LEfSe was used to generate a cladogram to identify the specific bacteria associated with different feeding models (Figure 5). In addition to the above-mentioned differential taxa, the GYG also showed significantly higher abundances of *Humicola, Vishniacozyma, Rachicladosporium*, and *Alternaria* as compared to the WYG and HFG.

**Table 1:** Statistical analysis of differential fungi between different groups.

**Taxa**	**WYG (%)**	**HFG (%)**	**GYG (%)**
Basidiomycota	28.61^a^	5.47^b^	5.86^b^
Ascomycota	69.90^b^	92.02^a^	90.18^a^
Mucoromycota	0.059^b^	0.58^a^	0.11^b^
Thelebolus	54.27^b^	84.52^a^	31.22^ab^
Cystobasidium	0.12^ac^	0.00080^b^	0.22^a^
Naganishia	25.43^a^	3.08^b^	1.71^b^
Cercophora	0.00^b^	0.14^a^	0.016^ab^
Stropharia	0.00^b^	0.014^a^	0.00^b^
Sclerostagonospora	0.095^a^	0.00^b^	0.00^b^
Lichtheimia	0.00^b^	0.065^a^	0.00^b^
Physalospora	0.077^a^	0.00^c^	0.015^b^
Neoascochyta	0.91^a^	0.00^c^	0.10^b^
Recurvomyces	0.077^a^	0.00^b^	0.071^ab^
Schizonella	0.019^a^	0.00^b^	0.0099^ab^
Rhizopus	0.019^b^	0.12^a^	0.032^ab^
Orpinomyces	0.021^a^	0.00^b^	0.059^ab^
Herpotrichia	0.0049^a^	0.00^b^	0.00^b^
Sphaerulina	0.0043^a^	0.00^b^	0.012^a^
Podospora	0.0098^a^	0.00^b^	0.055^ab^
Cercozoa	0.012^b^	0.00^b^	0.059^a^
Didymella	0.085^b^	0.14^ab^	0.25^a^
Claussenomyces	0.050^b^	0.046^b^	0.33^a^
Kurtzmanomyces	0.013^a^	0.022^b^	0.00^b^
Anthracoidea	0.074^b^	0.12^b^	0.37^a^
Papiliotrema	0.14^b^	0.056^b^	1.00^a^
Mastigosporium	0.00^b^	0.00^b^	0.022^a^
Dioszegia	0.0099^b^	0.00^b^	1.34^a^
Thermoascus	0.049^a^	0.039^ab^	0.0014^b^
Heyderia	0.00^b^	0.00^b^	0.041^a^
Ramularia	0.00^b^	0.00^b^	0.28^a^
Cladonia	0.00^b^	0.00^b^	0.024^a^
Urocystis	0.0075^b^	0.00^b^	0.11^a^
Panaeolus	0.0041^a^	0.00^b^	0.00^b^
Archaeorhizomyces	0.044^a^	0.10^ab^	0.00^b^
Parapenidiella	0.00^b^	0.00^b^	0.17^a^
Saitozyma	0.022^ab^	0.13^a^	0.00^b^
Mycosphaerella	0.20^ab^	0.00^b^	0.40^a^
Penicillium	0.073^ab^	0.11^a^	0.013^b^
Pyrenochaetopsis	0.056^ab^	0.058^a^	0.00^b^
Thermomyces	0.0038^ab^	0.094^a^	0.00^b^
Humicola	0.012^ab^	0.00^b^	0.19^a^
Knufia	0.0083^ab^	0.027^a^	0.00^b^
Melanocarpus	0.00^b^	0.044^a^	0.00^b^

Different letters in the same row indicate significant differences from each other.

## Discussion

The wild yak is an endangered breed that has been listed as a first-class protected wild animal in China. Compared with WYG, domestic yaks can be artificially intervened and raised. Currently, there are two main feeding modes for domestic yaks: free range and artificial breeding. Growing studies have indicated that the gut fungal community plays a role in host health, metabolism, and the immune system (7, 11, 42). Recent studies on the gut fungal community have also revealed its important driving roles in the development of diarrhea, inflammatory bowel disease, and liver cirrhosis (3, 43). Currently, studies of the gut fungal community have involved many species, such as mice, pigs, and dairy cattle, and revealed their importance in host health and development (19, 39, 44). However, research on the characteristics and differences of the gut fungal community in yaks under different feeding models is still insufficient to date. Analyzing the gut microbiota of yaks under different feeding models may contribute to revealing the differences in various characteristics of these yaks. In this study, we dissected the compositions and differences of the gut fungal community among WYG, HFG, and GYG.

Microbes that inhabit the intestine can interact in symbiotic or antagonistic relationships, which are an important basis for the maintenance of gut microbial homeostasis (45, 46). Gut microbial homeostasis is the precondition for maintaining host metabolism, digestion, and absorption (47, 48). Inversely, the disruption of gut microbial homeostasis may affect intestinal barrier function and immune function, seriously threatening host health (49, 50). Gut microbial homeostasis could be assessed by diversity indices such as Chao1, ACE, Shannon, and Simpson. Typically, the diversity index representing gut microbial homeostasis is in a relatively stable state due to the plasticity of the gut microbiota (51, 52). However, multiple factors, such as exercise, diet, age, and environmental pollutants, can affect the composition and structure of the gut microbiota, thereby affecting gut microbial homeostasis (53–55). In this study, we observed that the gut fungal Shannon and Simpson indices of WYG and GYG were higher than those of HFG, indicating that WYG and GYG have higher microbial diversity. Early surveys indicated that the higher diversity represents stronger gut microbial plasticity, which contributes to improving the gut microbiota's response to external stress, thereby maintaining gut microbial homeostasis (56, 57). Furthermore, increased gut microbial diversity also contributes to the maintenance and improvement of intestinal functions such as energy utilization and nutrient intake (58, 59). Studies have shown that primitive yaks existed in the Pleistocene more than 3 million years ago and were widely distributed in northeast Eurasia. Later, primitive yaks moved south to the Tibetan Plateau of China due to crustal movement and climate change, adapted to the Alpine climate, and continued to evolve into modern yaks. WYG inhabits plateau meadows, shrubs, and deserts at an altitude of 4,000 to 5,000 meters, which have strong adaptability to the environmental conditions of alpine grasslands. Environmental factors, such as altitude hypoxia and low temperatures, are important players in the evolution of gut microbiota (60, 61). Compared with domestic yaks, WYGs inhabit higher altitudes and harsher living environments. Therefore, WYG may have evolved a more complex gut microbial structure to adapt to complex environments. Compared with HFG, WYG, and GYG will constantly migrate in search of food and water supplies, which inevitably increases the amount of exercise for yaks. Numerous studies have indicated that the frequency of exercise is closely related to gut microbial composition and structure (62, 63). For instance, physical exercise could significantly alter the composition and diversity of the gut microbiota in mice (54). Compared with HFG, WYG, and GYG have more complicated and irregular diets. In addition, some plants in the wild environment may be polluted or contain pathogenic microorganisms, which also contribute to the evolution of a more complex gut microbial community. Therefore, we speculated that exercise, complex diet, and harsh environment may be some of the reasons for the more diverse gut microbiota of WYG and GYG.

The gut fungal community, as a vital constituent of the gut microbiota, is critical for host health (64, 65). The gut fungal community has been shown to be involved in the development of intestinal barrier function and intestinal inflammation (14, 66). Furthermore, recent investigations of the gut fungal community have also provided evidence that it is closely associated with diarrhea (3, 34). However, the importance and role of the gut fungal community in the host have long been overlooked because of their low proportion in the gut microbiota. To further explore the effects of different feeding models on the yaks, we also assessed the composition and variability of the gut fungal community in these populations. The results demonstrated that the phyla Ascomycota and Basidiomycota were abundantly present in the wild yak, HFG, and GYG, indicating that the different feeding models could not affect the species of the main dominant phyla. Moreover, Ascomycota and Basidiomycota have also been demonstrated to be major dominant fungal phyla in other animals, such as giraffes, sheep, and cows, suggesting their importance in the ruminant fungal community and intestinal function (3). Notably, although the species of the main dominant fungal phyla did not change, their abundances did. For instance, the abundance of Basidiomycota was significantly higher in WYG than in GYG and HFG. Basidiomycota is the highest phylum of fungi with more than 20,000 species, characterized by wide distribution, large number, and variety (67). Among these three types of yaks, WYG possessed the most complicated habitat environment and diet structure, which may change the gut microbial composition of yaks. Meanwhile, this may also be one of the reasons for the increase in Basidiomycota in WYG. In this study, we also observed significant changes in the abundance of some fungal genera between different feeding models. Previous investigations showed that shifts in some specific microbial communities could affect host phenotypes and intestinal functions (34, 68). The ecological environment and dietary structure are important driving factors for gut microbial succession, and the gut microbiota will change appropriately under various external stimuli to adapt to the habitat environment.

## Conclusions

This research explores the dynamic changes of the gut fungal community in yaks under different feeding models. The results indicated that feeding models could significantly alter the gut fungal composition and structure of yaks, including significant changes in some dominant phyla (Ascomycota and Basidiomycota) and fungal genera (*Thelebolus, Naganishia*, and *Vishniacozyma*). Moreover, WYG and GYG had a higher gut fungal diversity compared to HFG. This investigation elucidated the characteristics of the gut fungal community in WYG, HFG, and GYG and filled a gap in the effects of different feeding models on the gut fungal community. Meanwhile, this research also indicated that different feeding models are important drivers of changes in the gut fungal community. However, this study has some limitations that need to be acknowledged, such as the small sample size and lack of information on age and dietary habits.

## Data availability statement

The datasets presented in this study can be found in online repositories. The names of the repository/repositories and accession number(s) can be found at: https://www.ncbi.nlm.nih.gov/, PRJNA948534.

## Ethics statement

The study was conducted under the guidance and approval of the Animal Welfare and Ethics Committee of Institute of Animal Husbandry and Veterinary Medicine, Tibet Academy of Agriculture and Animal Husbandry Sciences.

## Author contributions

YZ and WB provided the research idea. YC, GS, XL, ZL, LS, and ZS contributed reagents, materials, and analytical tools. YZ wrote the manuscript. MS, YZ, and WB revised the manuscript. All authors participated in the writing and review of the manuscript.

## Appendix

**Appendix A:** Fungal sequence information for each sample.

**Sample**	**Effective reads**
WYG1	30,248.00
WYG2	31,083.00
WYG3	42,954.00
WYG4	35,967.00
WYG5	41,982.00
HFG1	38,727.00
HFG2	37,160.00
HFG3	41,853.00
HFG4	39,301.00
HFG5	36,407.00
GYG1	45,539.00
GYG2	42,840.00
GYG3	36,021.00
GYG4	32,081.00
GYG5	44,801.00
